# Decision-making of Adjuvant Chemotherapy for Breast Cancer Patients with Discordant Risk Classifications between Clinical-Pathological Factors and 21-gene Recurrence Score

**DOI:** 10.7150/jca.38976

**Published:** 2020-02-10

**Authors:** Weiqi Gao, Lin Lin, Xiaochun Fei, Xiaosong Chen, Kunwei Shen

**Affiliations:** 1 Comprehensive Breast Health Center, Ruijin Hospital, Shanghai Jiaotong University School of Medicine, Shanghai 200025, China; 2 Department of clinical laboratory, Ruijin Hospital, Shanghai Jiaotong University School of Medicine, Shanghai 200025, China; 3 Department of pathology, Ruijin Hospital, Shanghai Jiaotong University School of Medicine, Shanghai 200025, China

**Keywords:** breast cancer, Adjuvant! Online, 21-gene recurrence score, discordant risk, adjuvant chemotherapy

## Abstract

**Background**: Clinical-pathological factors and 21-gene recurrence score (RS) influence adjuvant chemotherapy (ACT) decision for early breast cancer patients. We investigated the decision-making of ACT in patients with discordant risk classifications of clinical-pathological factors and RS.

**Methods**: Patients with hormonal receptor (HR)+/ human epidermal growth factor receptor 2 (HER2)-, early breast cancer, who underwent 21-gene RS testing were identified from Ruijin Hospital (RJBC) and the Surveillance, Epidemiology, and End Results (SEER) database. According to Adjuvant! Online and RS (≤25 or >25), discordant risk classifications were defined as: clinical low-risk/ RS high-risk (C-low/ RS-high) and clinical high-risk/ RS low-risk (C-high/RS-low). McNemar's test was used to assess the changes between pre- and post-RS recommendations. Breast cancer-specific survival (BCSS) was estimated using the Kaplan-Meier methods.

**Results**: Among 727 RJBC patients, the C-low/RS-high group and the C-high/RS-low group represented 19.7% and 21.3% of the cohort. After receiving 21-gene RS results, treatment recommendations were changed for 22.1% patients with discordant risk classifications: ACT rate increased from 41.9% to 75.5% in the C-low/RS-high group and decreased from 63.9% to 60.0% in the C-high/RS-low group. Among 2958 patients from the SEER cohort, 18.4% of the C-high/RS-low group and 59.2% of the C-low/RS-high group received ACT. There was no significant difference in the estimated 3-year BCSS between ACT or not among the C-low/RS-high group (p=0.708) and the C-high/RS-low groups (p=0.391).

**Conclusion**: For patients with discordant risk classifications, physicians were apt to adopt the 21-gene RS rather than routine clinical-pathological factors to guide ACT selection.

## Introduction

More than half of female diagnosed with breast cancer have hormonal receptor (HR)+/ human epidermal growth factor receptor 2 (HER2)- early stage disease 1. According to current guidelines 2, 3, the primary treatment for these patients should include surgery with or without radiation therapy, followed by systemic treatment. In clinical practice, patients with HR+/HER2-, node-negative disease would be recommended to receive endocrine therapy with or without adjuvant chemotherapy (ACT) according to risk of recurrence 4. Considering the potential toxicity of ACT, the survival benefits of ACT must be weighed by accurately assessed disease outcome.

Before multi-gene expression signatures were developed, anatomic parameters (T and N) and histological parameters (Grade, ER, PR, Her-2 and Ki67) were used to guide adjuvant treatment selection. However, the need for individualized treatment urges novel methods to tailor therapies for early breast cancer. Multi-gene expression signatures have been increasingly developed to predict risk of recurrence and tumor response to ACT, which may also help clinicians to identify patients who were less likely to benefit from ACT 5-7. The omission of unnecessary ACT can spare patients from considerable side-effects and reduce health care costs. The most widely accepted gene expression signatures for breast cancer includes the 21-gene recurrence score (RS) and 70-gene signature, which can categorize patients into different risk groups. These assays have been validated to offer additional information that can refine the prognosis and ACT responsiveness among women with HR+/HER2-, lymph nodes negative (pN0) tumors in retrospective and prospective studies 8-11. Based on previous studies, the American Society for Clinical Oncology (ASCO) and St. Gallen International Expert Consensus have issued recommendations for the use of gene expression signatures in ACT decision-making among women with HR+/HER2- breast cancer 2, 3.

With the additional prognostic and predictive information from multi-gene expression assay, some decision-making of ACT would become more complex when patients were classified into discordant risk classifications by traditional biomarkers and multi-gene expression assay. In the MINDACT study 11, 8.8% of patients were defined as low clinical risk but high genomic risk by 70-gene signature and Adjuvant Online!, while 23.2% were high clinical risk but low genomic risk. Among these patients with discordant risk classifications, no significant difference of DMFS or DFS between the ACT group and the no ACT group was observed, which indicates multi-gene expression assay can be used in combination with traditional biomarkers in ACT decision-making, to preclude ACT for patients with high clinical risk but low 70-gene risk patients. However, although the updated results from TAILORx study 12 have confirmed the clinical utility of RS, there was no evidence exploring the value of RS when combined with Adjuvant Online! in the management of early breast cancer.

Therefore, the aims of the current study were to investigate whether 21-gene RS assay would influence ACT decision-making when added to traditional clinical-pathological factors, particularly among women with discordant risk classifications, and then to study its impact on patients' adherence to treatment recommendation.

## Materials and Methods

### Patients and clinical-pathological data

Women treated in Comprehensive Breast Health Center, Ruijin Hospital from January 2014 to August 2018 were included as the RJBC cohort. Patients between 18 and 75 years old with histologically proven HR-positive, HER2-negative invasive breast cancer and tested with 21-gene RS were recruited. Main exclusion criteria included male, pT1a or pT4 disease, pN2-3, synchronous bilateral breast cancer, metastatic breast cancer, previous or concurrent malignant disease, and neo-adjuvant systemic therapy for breast cancer.

Patient and tumor characteristics of the RJBC cohort were obtained from Shanghai Jiao Tong University Breast Cancer Database (SJTU-BCDB), including age, menopausal status, type of surgery, pathology, grade, expression level of ER, PR, Ki67, TNM stage, 21-gene RS, actual adjuvant treatment, and follow-up information. Multi-disciplinary team (MDT) recommendations for adjuvant treatment were collected from MDT4BC System in Comprehensive Breast Health Center, Ruijin Hospital.

A second cohort was collected from the Surveillance, Epidemiology, and End Results (SEER) database between 2010 to 2014^13^. The eligibility criteria for the SEER cohort included: (1) female, (2) histologically proved ER-positive, HER2-negative invasive breast cancer, (3) pT1-2N0M0 disease and (4) RS available. Main exclusion criteria included: (1) synchronous bilateral breast cancer, (2) metastatic breast cancer, (3) previous or concurrent malignant disease, and (4) neo-adjuvant systemic therapy for breast cancer.

Patient and tumor characteristics obtained from the SEER database included: age at diagnosis, race, pathological tumor stage, and histological subtype, status of HR and HER2, tumor grade, and basic treatment information such as surgery type, radiation therapy, and chemotherapy. Information regarding chemotherapy in the SEER database was classified into two groups: (1) yes and (2) no/unknown. Those patients with no ACT information was considered as receiving no ACT in our study. And RS was also obtained on our request.

The current study was approved by independent ethics committees of Ruijin Hospital and the study conforms to recognized standards. For patients of the RJBC cohort, they gave their written informed consent prior to their inclusion in the study. The results of this study do not affect the treatment decision of those patients enrolled. And the SEER cohort was exempted from the independent ethics committees of Ruijin Hospital, because of its anonymous clinical-pathological information.

### Risk classification categories

Of the RJBC cohort, patients were categorized into clinical low risk (C-low) and clinical high risk (C-high) according to Adjuvant! Online (version 8.0, www.adjuvantonline.com) 11. And for the SEER cohort, due to the lack of the exact tumor size, C-low was defined as pT1 and grade I-II disease, C-high as pT2 & grade II-III disease.

Genomic risk is defined by 21-gene RS results. RS was determined from FFPE tissue as previously described. In brief, expression of 16 cancer genes was measured in triplicate, and normalized relative to a set of five reference genes 5. According to the TAILORx study results 12, patients with RS≤25 were defined as RS low risk (RS-low), and patients with RS>25 were defined as RS high risk (RS-high) for both cohorts.

### Study design

In the RJBC cohort, patients would undergo a consultation for adjuvant treatment with the multi-disciplinary team (MDT) after surgery, which consisted of surgical oncologists, medical oncologists, radiologists, pathologists, and breast care nurses. With traditional clinical-pathological factors, preliminary recommendation for or against ACT was made and recorded in the MDT4BC System as patients' pre-RS recommendation. After initial MDT, 21-gene assay was then administered when appropriate. After RS results were available, patients would undergo a second-round consultation with MDT. Based on all information, MDT would make a final treatment recommendation regarding ACT, which was recorded as post-RS recommendation in the MDT4BC System. Patients' adherence rate to post-RS recommendation among patients with discordant risk classifications were also analyzed.

The SEER cohort was used to evaluate the rate of ACT across two discordant risk groups and investigated the impact of ACT on disease outcome.

### Statistical analyses

All statistical analyses were carried out in SPSS version 18.0. McNemar's test was used to assess whether the changes between pre- and post-RS recommendations was significant. Univariate and multivariate logistic regression analysis were used to assess the association between clinical-pathological or genomic factors and post-RS recommendation. Breast cancer-specific survival (BCSS) was defined as the time of diagnosis of breast cancer to the time of death from breast cancer. Patients without events were censored at the time of last follow-up. BCSS was estimated using the Kaplan-Meier estimator and tested with log-rank tests. All statistical tests were 2 tailed and considered significant for P <0.05.

## Results

### The RJBC Cohort

#### Baseline Characteristics

**Figure 1:**
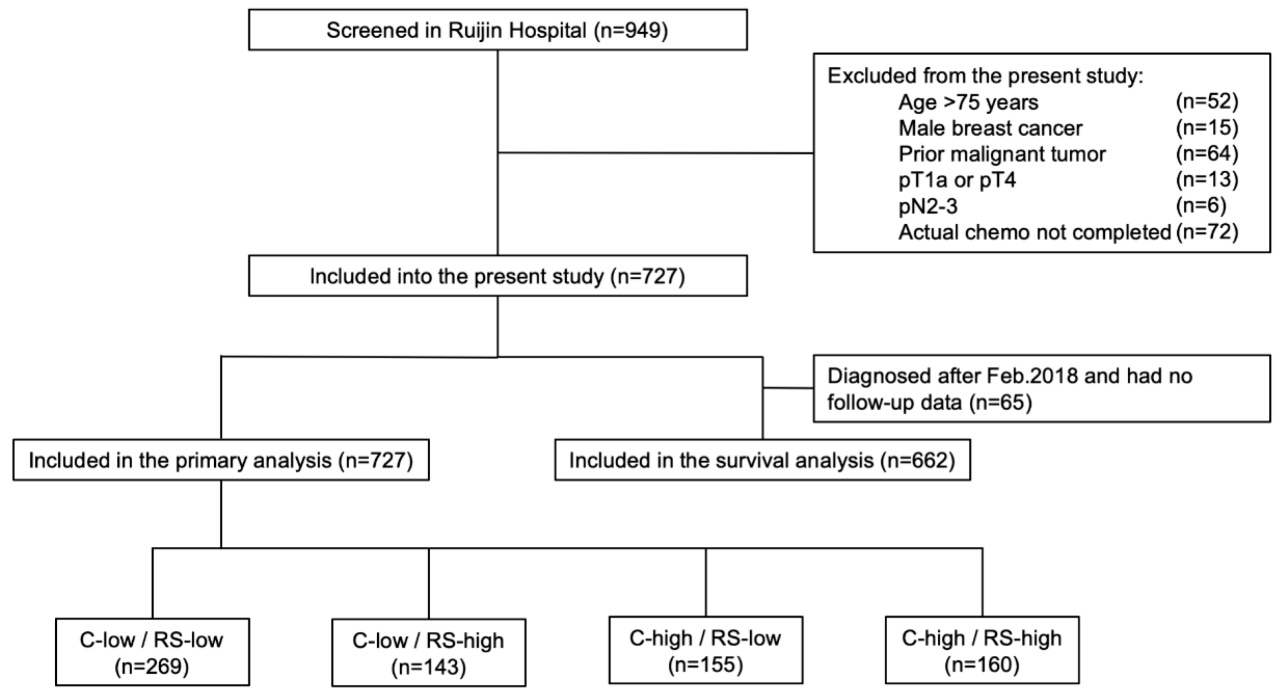
Study flow chart, RJBC 2014-2018 (n=727). Abbreviations: C-low: clinical low-risk; C-high: clinical high-risk; RS-low: recurrence score low-risk; RS-high: recurrence score high-risk; RS: recurrence score.

Among 949 patients enrolled from January 2014 to August 2018, 727 patients were included into the final cohort (Fig 1). Distributions of main characteristics were listed in Table 1. Median age was 58 years, with 21.7% of patients (n=158) >65 years, while 33.0% were pre- or peri-menopausal. There were 69.2% patients with pT1 and 30.8% with pT2-3 disease. Regarding lymph node status, 81.4%, 2.9%, and 15.7% patients were diagnosed as pN0, pNmic, and pN1, respectively. Grade was stratified into grade I-II and grade III, representing 80.2% and 19.8% of the patients. Rate of luminal-B like tumor was 70.2%, whereas 29.8% had luminal-A like disease.

Based on Adjuvant! Online and RS, enrolled patients were categorized into four groups: the C-low/RS-low group (269 patients, 37.0%), the C-low/RS-high group (143 patients, 19.7%), the C-high/RS-low group (155 patients, 21.3%), and the C-high/RS-high group (160 patients, 22.0%).

#### MDT's recommendation before and after RS

In the initial consultation without 21-gene RS, 52.3% of the patients were recommended with ACT, and 47.7% recommended against ACT. Among the 412 patients in the C-low group, ACT was recommended for 29.4% of patients, while the proportion was 75.0% among patients in the C-high group.

**Figure 2:**
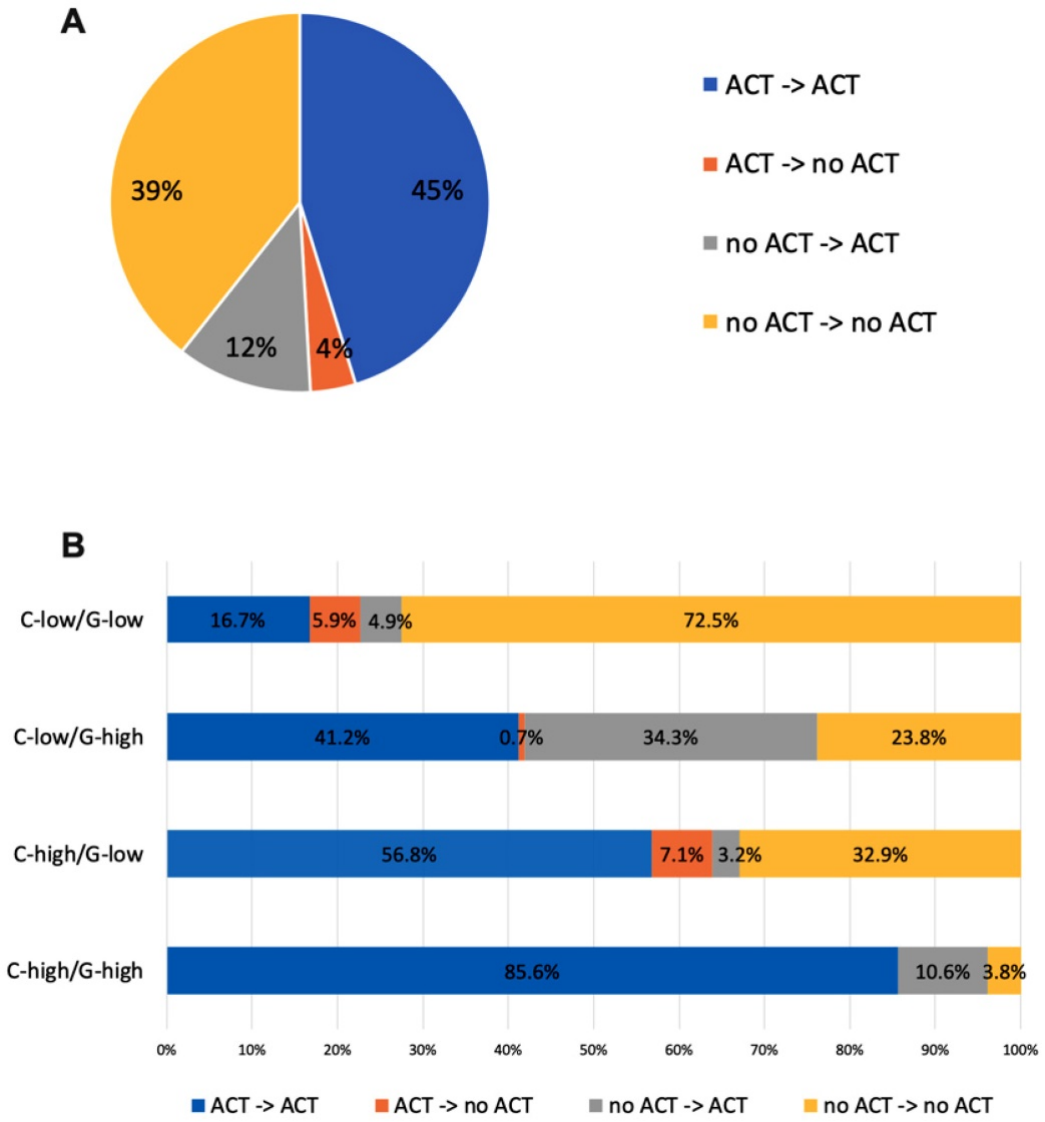
Treatment recommendation changes before and after RS of the RJBC cohort (A) in the whole population, (B) in patients with different risk groups. Abbreviations: C-low: clinical low-risk; C-high: clinical high-risk; RS-low: recurrence score low-risk; RS-high: recurrence score high-risk; ACT: adjuvant chemotherapy; RS: recurrence score.

With knowledge of RS, 22.1% of patients with discordant risk classifications have their post-RS recommendation changed compared to pre-RS recommendation, while the percentage was only 10.7% among patients with consistent risk classification (P<0.001). Table 2 showed the number of patients recommended ACT in each risk group pre and post-RS. After 21-gene results, recommendations switched from no ACT to ACT for 34.3% of patients in the C-low/RS-high group, and from ACT to no ACT for 0.7%. For patients with C-high/RS-low, recommendation changed to ACT for 3.2% patients and changed to no ACT for 7.1% patients (Figure 2).

Among patients with discordant risk classifications, recommendation of ACT was significantly correlated with 21-gene RS results: 75.5% of patients with RS>25 were recommended ACT, while 60% of patients with RS≤25 were recommended ACT (P=0.005). Multivariable analysis found that high RS was independently associated with post-RS recommendation for ACT (OR=5.48, 95%CI 2.27-13.21, P<0.001). Other independent risk factors for recommending ACT were special pathology types (versus IDC, OR=0.11, 95%CI 0.04-0.29, P<0.001), nodal involvement (OR=10.78, 95%CI 4.23-17.51, P<0.001), high expression of PR (OR=0.22, 95%CI 0.09-0.51, P<0.001), and high expression of Ki67 (OR=4.50, 95%CI 2.34-8.65, P<0.001) (Table 3).

#### Actual treatment

In the whole population, 95.6% of patients followed post-RS recommendation. Among the 413 patients with a post-RS recommendation for ACT, 386 (93.5%) patients received ACT. Meanwhile, among the 314 patients recommended no ACT, there were 309 (98.4%) patients spared from ACT in actual treatment.

Among 298 patients with discordant risk classifications, 284 patients (95.3%) followed post-RS recommendation regarding ACT. In detail, 10 patients (3.4%) refused to receive recommended ACT while 4 patients (1.3%) were treated with ACT irrespective of no ACT recommendation. And for the 170 patients whose post-RS recommendations were in line with RS results (ACT if RS>25, no ACT if RS≤25), 164 patients (96.5%) actually followed post-RS recommendation. Whereas for 120 patients whose post-RS recommendations were in line with clinical risk (ACT if C-high, no ACT if C-low), 120 patients (93.8%) actually followed post-RS recommendation. (P=0.284).

#### Survival outcomes

There were 65 patients (8.9%) diagnosed after February 2018 without follow-up information, thus a total of 662 patients were included for survival analysis. The median follow-up time was 18.5 months. Among 281 patients with discordant risk classifications, 2 cases with distant metastasis and 2 cases with second primary cancer were observed in patients receiving ACT, while 1 case with distant metastasis was detected in patients not receiving ACT.

### The SEER cohort

#### Baseline Characteristics

From 2010 to 2014, a total of 31,575 patients were reviewed from the SEER database: 2196 patients with C-high/RS-low and 762 patients with C-low/RS-high disease. Distributions of main characteristics were listed in Table 4. Median age was 59 years old, ranging from 19-91. There were 25.8% patients with pT1 disease and 74.2% with pT2 disease. Grade was stratified into grade I-II, and grade III, representing 84.3%, and 15.7% of the cohort. All tumors were ER-positive and HER2-negative, while 89.0% tumors were PR-positive. Breast conserving surgery was performed in 65.6% of the cohort, and radiation therapy was undergone in 58.9% patients.

#### Adjuvant Chemotherapy

**Figure 3:**
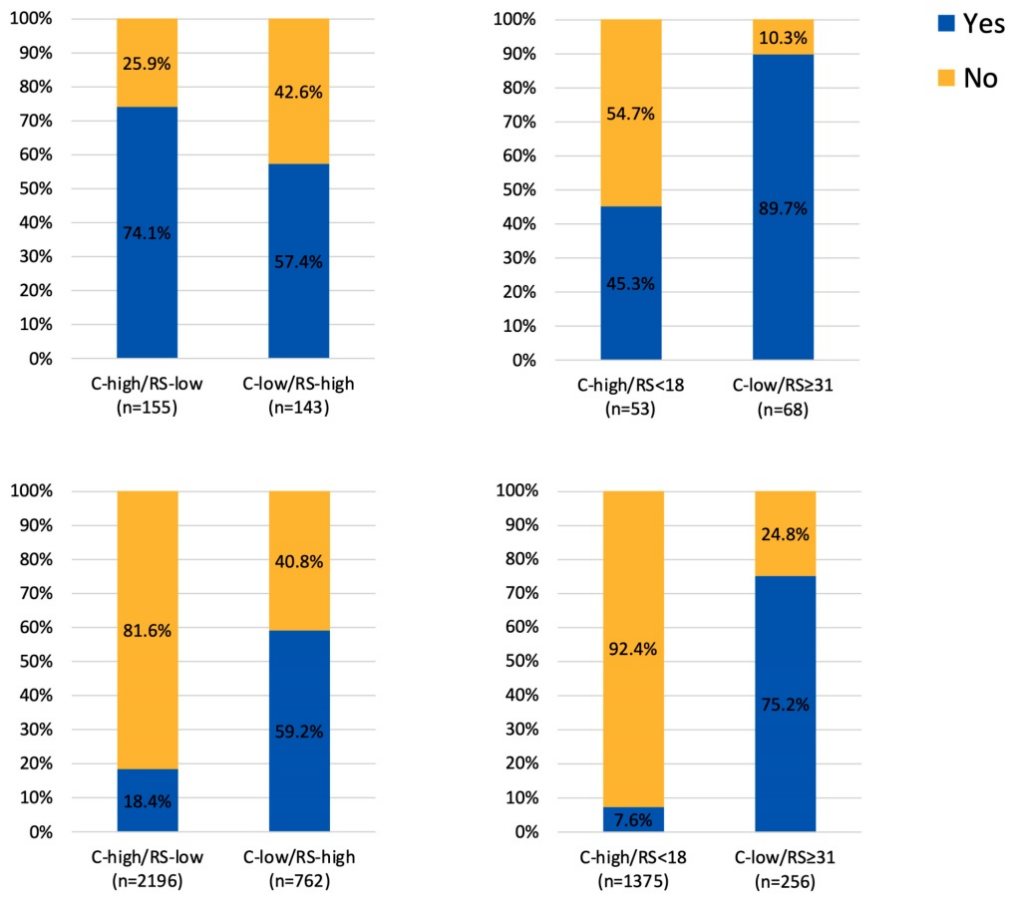
Adjuvant chemotherapy usage in patients with discordant risk classifications (A) 25 as cutoff value for RS in the RJBC cohort, (B) 18 and 31 as cutoff value for RS in the RJBC cohort, (C) 25 as cutoff value for RS in the SEER cohort, (D) 18 and 31 as cutoff value for RS in the SEER cohort

Regarding the ACT, there were 405 patients (18.4%) in the C-high/RS-low group receiving ACT, while 311 patients (59.2%) in the C-low/RS-high group received ACT (Figure 3). In the exploratory analysis, we further took 18 and 31 as the cutoff value for RS risk category classification. There were 1375 patients with C-high/RS<18 low disease and 256 patients with C-low/RS≥31 disease. Regarding the ACT, 7.6% in the C-high/RS-low group and 75.2% in the C-low/RS-high group have received ACT (Figure 3).

#### Survival Outcome

**Figure 4:**
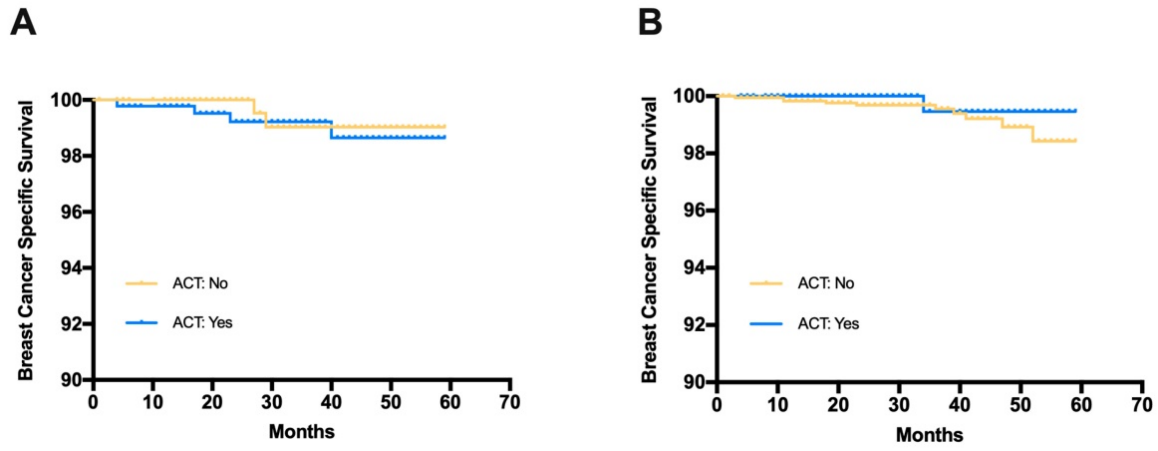
Breast cancer specific survival (BCSS) in patients receiving adjuvant chemotherapy or not (A) Patients with C-low/RS-high risk from the SEER database, (B) Patients with C-high/RS-low risk from the SEER database

The median follow-up time for the SEER cohort was 33 months. Among patients with C-low/RS-high disease, the estimated 3-year BCSS was 99.2% in the ACT group and 99.0% in the no ACT group (Fig 4). The log-rank test suggested that there was no significant difference in BCSS between the ACT group and the no ACT group (HR=1.365, 95%CI 0.268-6.953, p=0.708). Similar results were observed in patients with C-high/RS-low disease (HR=0.418, 95%CI 0.116-2.32, p=0.391). The estimated 3-year BCSS was 99.5% in the ACT group and 99.6% in the no ACT group for C-high/RS-low patients.

## Discussion

Our study included a cohort of 727 patients with pre-RS and post-RS ACT recommendations and demonstrated the real-world impact of 21-gene RS on MDT's decision-making, especially in patients with discordant risks of clinical-pathological factors and 21-gene RS. For those patients with discordant risk classification, 54 patients (18.4%) had their post-RS recommendation switched to ACT, whereas only 12 patients (4.0%) had their post-RS recommendation switched to no ACT. Agreement between patients' actual treatment and MDT's post-RS recommendation was achieved with 95.3% in the discordant risk cohort. Furthermore, a second cohort from the SEER database showed that physicians were apt to adopt the 21-gene RS rather than routine clinical-pathological factors to guide ACT decision, with a significantly higher rate of ACT in the C-low/RS-high group compared with the C-high/RS-low group.

Traditionally, anatomic staging and histological parameters (Grade, ER, PR, Her-2 and Ki67) were the mainstay to predict disease outcome and guide adjuvant treatment for HR+/HER2- early breast cancer. Based on several clinical studies 5, 6, 9, 12, multigene expression assay has now been incorporated into clinical practice with traditional prognostic biomarkers for adjuvant treatment in HR+/HER2- early breast cancer. However, results from the previous trials have also indicated the potential of inconsistent results between clinical-pathological factors and multi-gene expression assay results. In the MINDACT trial, a total of 2142 patients out of the 6693 enrolled patients has discordant risk classifications by Adjuvant! Online and 70-gene signature. In our center, by using 21-gene RS instead of 70-gene signature, we found similar discordant rate between clinical and 21-gene RS result, with 19.7% patients in the C-low/RS-high and 21.3% in the C-high/RS-low group.

The prospective TAILORx trial 12 and WSG Plan B trial 14 have shown the predictive value of RS on ACT in HR+/HER2- early breast cancer, supporting that 21-gene RS can be incorporated into traditional clinical-pathological factors for ACT decision-making. Based on these findings, studies observed changes of clinicians' treatment recommendation after 21-gene RS testing in clinical practice, such as the prospective PONDx study, which found recommendation change in 44% of the study cohorts and a reduction of 36% in ACT recommendation 15. In the RJBC cohort of our study, we found that MDT's treatment recommendation was influenced by 21-gene RS. MDT's recommendation change rate was 16.0% in the whole population and 22.1% in patients with discordant risk classifications. Inconsistent with previous studies, our MDT's recommendations change towards more ACT: with an increase of ACT recommendation in 34.3% of patients in the C-low/RS-high group and a decrease of ACT recommendation in 7.1% of patients in the C-high/RS-low group, leaving 56.8% patients still recommended to receive ACT. This indicated that physicians were more willing to suggest ACT in patients with high RS but hesitated to withdraw chemotherapy for patients with low RS. Within the SEER cohort, we found that patients in the C-low/RS-high group had more probability to receive ACT compared with patients in the C-high/RS-low group. When we chose 18 and 31 as the cutoff for RS risk classification, the difference of ACT rates between two groups was more significant. The relative low rate of ACT in patients with C-high/RS-low indicated that physicians were apt to adopt the 21-gene RS rather than routine clinical-pathological factors to guide ACT selection.

The prospective MINDACT study 11 investigated the clinical value of 70-gene signature and Adjuvant! Online in guiding ACT for HR+/HER2- breast cancer patients, and concluded that patients with either clinically low risk or genomic low risk could be safely spared from ACT. Exploratory analysis from the TAILORx study found additional prognostic value of clinical risk classification to RS, while the predictive value on absolute ACT benefit was only restricted in patients age ≤ 50 and RS 16-25 16. Along with the previous results from 70-gene signature and 21-gene RS, the SEER cohort in our study also found no significant survival benefit of ACT among patients in the discordant risk group. And for the RJBC cohort with a median follow-up of 18 months, disease outcome was good for patients in the discordant risk group irrespective to receiving ACT or not.

Our current study has several limitations. Firstly, after TAILORx study result was released, the optimal cutoff value of RS for risk classification has changed 9, which may cause different risk classification based on the same 21-gene RS result. Secondly, information regarding ACT in the SEER database was classified into two groups: (1) yes and (2) no/unknown. Those patients with no ACT information was considered as receiving no ACT in our study, which may decrease the rate of ACT. Furthermore, the median follow-up time of the two cohorts was too short to observe disease outcome for HR+/Her2- breast cancer. So further study with prolonged follow-up time was warranted to evaluate the survival benefit of ACT in patients with discordant risk classification.

## Conclusion

Our study has shown that patients may be classified into discordant risk groups when incorporating 21-gene RS into traditional clinical-pathological factors. As risk of recurrence was inconsistent by clinical and genomic risk model, physicians were more attempted to make ACT decision based on the 21-gene RS rather than routine clinical-pathological factors.

## Figures and Tables

**Table 1:** Distributions of patient and tumor characteristics in whole population and different risk group, RJBC 2014-2018 (n=727)

Characteristics	Risk classification				All patients
	C-low/RS-low	C-low/RS-high	C high/RS-low	C high/RS-high	
Number of patients (%)	269 (37.0%)	143 (19.7%)	155 (21.3%)	160 (22.0%)	727 (100.0%)
Age, median (range), years	56 (28-75)	57 (30-75)	60 (27-75)	58 (27-75)	58 (27-75)
Menopausal Status, No. (%)					
pre-/peri-menopausal	111 (41.3%)	45 (31.5%)	41 (26.5%)	43 (26.9%)	240 (33.0%)
post-menopausal	158 (58.7%)	98 (68.5%)	114 (73.5%)	117 (73.1%)	487 (67.0%)
Surgery Type, No. (%)					
Mastectomy	149 (55.4%)	60 (42.0%)	106 (68.4%)	103 (64.4%)	418 (57.5%)
Breast conserving surgery	120 (44.6%)	83 (58.0%)	49 (31.6%)	57 (35.6%)	309 (42.5%)
Tumor Stage, No. (%)					
pT1	250 (92.9%)	138 (96.5%)	57 (36.8%)	58 (36.2%)	503 (69.2%)
pT2-3	19 (7.1%)	5 (3.5%)	98 (63.2%)	102 (63.8%)	224 (30.8%)
Nodal Status, No. (%)					
pN0-mic	263 (97.8%)	143 (100.0%)	92 (59.4%)	117 (73.1%)	615 (84.6%)
pN1	6 (2.2%)	0 (0.0%)	63 (40.6%)	43 (26.9%)	112 (15.4%)
Pathologic type, No. (%)					
IDC	236 (87.7%)	124 (86.7%)	144 (92.9%)	153 (95.6%)	657 (90.4%)
Others	33 (12.3%)	19 (13.3%)	11 (7.1%)	7 (4.4%)	70 (9.6%)
Tumor grade, No. (%)					
I-II	265 (98.5%)	139 (97.2%)	104 (67.1%)	75 (46.9%)	583 (80.2%)
III	4 (1.5%)	4 (2.8%)	51 (32.9%)	85 (53.1%)	144 (19.8%)
ER expression, No. (%)					
<50%	2 (0.7%)	7 (4.9%)	0 (0.0%)	12 (7.5%)	21 (2.9%)
≥50%	267 (99.3%)	136 (95.1%)	155 (100.0%)	148 (92.5%)	706 (97.1%)
PR expression, No. (%)					
<20%	39 (14.5%)	57 (39.9%)	16 (10.3%)	73 (45.6%)	185 (25.4%)
≥20%	230 (85.5%)	86 (60.1%)	139 (89.7%)	87 (54.4%)	542 (74.6%)
Ki-67 expression, No. (%)					
≤14	156 (58.0%)	75 (52.4%)	53 (34.2%)	34 (21.2%)	318 (43.7%)
>14	113 (42.0%)	68 (47.6%)	102 (65.8%)	126 (78.8%)	409 (56.3%)
Intrinsic subtype, No. (%)					
Luminal A-like	127 (47.2%)	32 (22.4%)	45 (29.0%)	13 (8.1%)	217 (29.8%)
Luminal B-like	142 (52.8%)	111 (77.6%)	110 (71.0%)	147 (91.9%)	510 (70.2%)

Abbreviations: C-low: clinical low-risk; C-high: clinical high-risk; RS-low: recurrence score low-risk; RS-high: recurrence score high-risk; IDC: invasive ductal carcinoma; ER: estrogen receptor; PR: progestogen receptor.

**Table 2:** Recommendations for adjuvant treatment before and after 21-gene RS, and actual adjuvant treatment in the whole population and different risk groups, RJBC 2014-2018 (n=727)

Post-RS	pre-RS		pre-RS to post-RS changed (%)	actual treatment		Compliance rate (%)
	ACT	no-ACT		ACT	no-ACT	
Whole Population						
ACT	329	84	112/727 (15.41%)	386	27	695/727 (95.6%)
no-ACT	28	286		5	309	
C-low/RS-low						
ACT	45	13	29/269 (10.8%)	48	10	258/269 (95.9%)
no-ACT	16	195		1	210	
C-low/RS-high						
ACT	59	49	50/143 (35.0%)	102	6	133/143 (93.0%)
no-ACT	1	34		4	31	
C-high/RS-low						
ACT	88	5	16/155 (10.3%)	89	4	151/155 (97.4%)
no-ACT	11	51		0	62	
C-high/RS-high						
ACT	137	17	17/160 (10.6%)	147	7	153/160 (95.6%)
no-ACT	0	6		0	6	
Discordant risk						
ACT	147	54	66/298 (22.1%)	191	10	284/298 (95.3%)
no-ACT	12	85		4	93	

Abbreviations: C-low: clinical low-risk; C-high: clinical high-risk; RS-low: recurrence score low-risk; RS-high: recurrence score high-risk; ACT: adjuvant chemotherapy; RS: recurrence score.

**Table 3:** Univariate and multivariate analyses for ACT recommendation among patients with discordant risk classifications, RJBC 2014-2018 (n=727)

Variables		Univariate analysis			Multivariate analysis	
		OR (95% CI)	P		OR (95% CI)	P
Pathology (Special types vs. IDC)		0.17 (0.07-0.39)	<0.001		0.11 (0.04-0.29)	<0.001
Tumor stage (T2-3 vs. T1)		0.41 (0.25-0.68)	0.001		0.93 (0.43-2.02)	0.860
Nodal status (pN1 vs. pN0-mic)		3.59 (1.69-7.63)	0.001		10.78 (4.23-17.51)	<0.001
PR (≥20% vs. <20%)		0.29 (0.14-0.58)	<0.001		0.22 (0.09-0.51)	<0.001
Ki67 (≥14% vs. <14%)		2.02 (1.24-3.31)	0.005		4.50 (2.34-8.65)	<0.001
RS (>25 vs. ≤25)		2.06 (1.25-3.39)	0.005		5.48 (2.27-13.21)	<0.001

Abbreviations: IDC: invasive ductal carcinoma; PR: progestogen receptor; RS: recurrence score.

**Table 4:** Distributions of patient and tumor characteristics in whole population and different risk groups, SEER 2010-2014 (n=2958)

Characteristics	All patients	Risk classification		
		C-low/RS-high	C-high/RS-low	p value
Age, median (range), years	59 (19-91)	59 (25-88)	59 (19-91)	
Tumor Size, No. (%)				<0.001
pT1	762 (25.8%)	762 (100%)	0 (0%)	
pT2	2196 (74.2%)	0 (0%)	2196 (100%)	
Grade, No. (%)				<0.001
I-II	2494 (84.3%)	762 (100%)	1732 (78.9%)	
III	464 (15.7%)	0 (0%)	464 (21.1%)	
PR expression, No. (%)				<0.001
positive	2634 (89.0%)	547 (71.8%)	2087 (95.0%)	
negative	324 (11.0%)	215 (28.2%)	109 (5.0%)	
Surgery, No. (%)				<0.001
Mastectomy	1019 (34.4%)	195 (25.6%)	824 (37.5%)	
Breast conserving surgery	1939 (65.6%)	567 (74.4%)	1372 (62.5%)	
Radiation therapy, No. (%)				0.003
Received	1742 (58.9%)	484 (63.5%)	1258 (57.3%)	
Not received	1216 (41.1%)	278 (36.5%)	938 (42.7%)	

Abbreviations: C-low: clinical low-risk; C-high: clinical high-risk; RS-low: recurrence score low-risk; RS-high: recurrence score high-risk; PR: progestogen receptor.

## References

[1] Howlader N, Altekruse SF, Li CI (2014). US Incidence of Breast Cancer Subtypes Defined by Joint Hormone Receptor and HER2 Status.

[2] Harris LN, Ismaila N, McShane LM (2016). Use of Biomarkers to Guide Decisions on Adjuvant Systemic Therapy for Women With Early-Stage Invasive Breast Cancer: American Society of Clinical Oncology Clinical Practice Guideline. J Clin Oncol.

[3] Curigliano G, Burstein HJ, E PW (2017). De-escalating and escalating treatments for early-stage breast cancer: the St. Gallen International Expert Consensus Conference on the Primary Therapy of Early Breast Cancer 2017. Ann Oncol.

[4] Waks AG, Winer EP (2019). Breast Cancer Treatment: A Review. Jama.

[5] Paik S, Shak S, Tang G (2004). A multigene assay to predict recurrence of tamoxifen-treated, node-negative breast cancer. N Engl J Med.

[6] Buyse M, Loi S, van't Veer L (2006). Validation and Clinical Utility of a 70-Gene Prognostic Signature for Women With Node-Negative Breast Cancer. J Natl Cancer Inst.

[7] Filipits M, Rudas M, Jakesz R (2011). A New Molecular Predictor of Distant Recurrence in ER-Positive, HER2-Negative Breast Cancer Adds Independent Information to Conventional Clinical Risk Factors. Clin Cancer Res.

[8] Paik S, Tang G, Shak S (2006). Gene Expression and Benefit of Chemotherapy in Women With Node-Negative, Estrogen Receptor-Positive Breast Cancer. J Clin Oncol.

[9] Sparano JA, Gray RJ, Makower DF (2015). Prospective Validation of a 21-Gene Expression Assay in Breast Cancer. N Engl J Med.

[10] Bueno-De-Mesquita JM, Linn SC, Keijzer R (2009). Validation of 70-gene prognosis signature in node-negative breast cancer. Breast Cancer Res Treat.

[11] Cardoso F, van't Veer LJ, Bogaerts J (2016). 70-Gene Signature as an Aid to Treatment Decisions in Early-Stage Breast Cancer. N Engl J Med.

[12] Sparano JA, Gray RJ, Makower DF (2018). Adjuvant Chemotherapy Guided by a 21-Gene Expression Assay in Breast Cancer. N Engl J Med.

[13] Ding S, Wu J, Lin C (2019). Evaluation of the Incorporation of Recurrence Score into the American Joint Committee on Cancer Eighth Edition Staging System in Patients with T1-2N0M0, Estrogen Receptor-Positive, Human Epidermal Growth Receptor 2-Negative Invasive Breast Cancer: A Population-Based Analysis. Oncologist.

[14] Nitz U, Gluz O, Christgen M (2017). Reducing chemotherapy use in clinically high-risk, genomically low-risk pN0 and pN1 early breast cancer patients: five-year data from the prospective, randomised phase 3 West German Study Group (WSG) PlanB trial. Breast Cancer Res Treat.

[15] Curtit E, Vannetzel J-M, Darmon J-C (2019). Results of PONDx, a prospective multicenter study of the Oncotype DX® breast cancer assay: Real-life utilization and decision impact in French clinical practice. Breast.

[16] Sparano JA, Gray RJ, Ravdin PM (2019). Clinical and Genomic Risk to Guide the Use of Adjuvant Therapy for Breast Cancer. N Engl J Med.

